# Microglia influence immune responses and restrict neurologic disease in response to central nervous system infection by a neurotropic murine coronavirus

**DOI:** 10.3389/fncel.2023.1291255

**Published:** 2023-11-30

**Authors:** Amber Syage, Collin Pachow, Yuting Cheng, Vrushali Mangale, Kim N. Green, Thomas E. Lane

**Affiliations:** ^1^Department of Neurobiology and Behavior, University of California, Irvine, Irvine, CA, United States; ^2^Department of Molecular Biology and Biochemistry, University of California, Irvine, Irvine, CA, United States; ^3^Department of Pathology, University of Utah, Salt Lake City, UT, United States; ^4^Center for Virus Research, University of California, Irvine, Irvine, CA, United States

**Keywords:** microglia, coronavirus, neuroinflammation, demyelination, remyelination

## Abstract

Intracranial (i.c.) inoculation of susceptible mice with a glial-tropic strain of mouse hepatitis virus (JHMV), a murine coronavirus, results in an acute encephalomyelitis followed by viral persistence in white matter tracts accompanied by chronic neuroinflammation and demyelination. Microglia serve numerous functions including maintenance of the healthy central nervous system (CNS) and are among the first responders to injury or infection. More recently, studies have demonstrated that microglia aid in tailoring innate and adaptive immune responses following infection by neurotropic viruses including flaviviruses, herpesviruses, and picornaviruses. These findings have emphasized an important role for microglia in host defense against these viral pathogens. In addition, microglia are also critical in optimizing immune-mediated control of JHMV replication within the CNS while restricting the severity of demyelination and enhancing remyelination. This review will highlight our current understanding of the molecular and cellular mechanisms by which microglia aid in host defense, limit neurologic disease, and promote repair following CNS infection by a neurotropic murine coronavirus.

## 1 Introduction

The Coronaviridae family is composed of large (26–32 kb), enveloped, single-stranded RNA viruses in the order Nidovirales (Bergmann et al., 2006; Gorbalenya et al., 2006) that are classified into groups based on shared sequencing homologies and serologic cross-reactivity (Bergmann et al., 2006; Gorbalenya et al., 2006). Their naming dates to the 1960s when McIntosh et al. (1967) isolated infectious bronchitis virus (IBV)-like virions from the upper respiratory tracts of patients with common colds. When electron micrographs of avian IBV and the newly isolated human strains were compared, the “club- or pear-shaped” projections on their viral coats showed strikingly similar characteristics, which ultimately led to grouping these viruses together with the coronavirus family name, based on the Latin root, “corona,” for their crown-like appearance (Tyrrell and Myint, 1996).

The neuroattenuated John Howard Mueller (JHM) strain of mouse hepatitis virus (JHMV) is a well-characterized laboratory strain that can cause severe encephalomyelitis and demyelination in adult mice (Cheever et al., 1949; Fleming et al., 1986). Infection of susceptible mice with JHMV has proven to be an effective mouse model for studying various immunologic and pathologic features associated with viral-induced neurologic disease. Following a sublethal intracranial (i.c.) infection with JHMV, virus rapidly infects and replicates within ependymal cells lining the lateral ventricles (Wang et al., 1992; Lane et al., 1998). Within 24-h, JHMV rapidly spreads and penetrates further into the parenchyma where astrocytes, oligodendrocytes, and microglia are targets of infection, while neurons are relatively spared (Fleming et al., 1986; Wang et al., 1992). Viral titers within the brain peak between days 5 and 7 post-infection (p.i.) and decline below levels of detection by plaque assay (∼100 PFU/g tissue) between 10 and 14 days p.i. Sterilizing immunity is incomplete, as viral antigen and RNA are capable of persisting within the CNS (Marten et al., 2000), and this is associated with chronic neuroinflammation leading to an immune-mediated demyelinating disease, which is mediated by inflammatory T cells and myeloid cells (Perlman et al., 1999; Wu and Perlman, 1999; Wu et al., 2000; Glass et al., 2001, 2004; Liu et al., 2001c; Dufour et al., 2002; Pewe and Perlman, 2002; Pewe et al., 2002; Kim and Perlman, 2005; Bergmann et al., 2006; Sariol et al., 2020). More recently, roles for microglia in both demyelination and remyelination in JHMV-infected mice have been implicated (Wheeler et al., 2018; Mangale et al., 2020; Sariol et al., 2020). Due to the clinical and histologic similarities to the human demyelinating disease, multiple sclerosis (MS), the JHMV model of viral-induced neurologic disease is considered a relevant pre-clinical model of MS (Lane and Buchmeier, 1997; Bergmann et al., 2006).

## 2 Microglia

Microglia are the brain’s resident immune cells, comprising approximately 5–12% of all cells found in the brain, and, together with perivascular, choroid plexus, and meningeal macrophages, comprise the macrophage compartment of the CNS. Originally presumed to be “resting,” microglia in the healthy adult brain are highly dynamic, surveying the entire brain parenchyma every 24 h. In this “surveying” state, microglia exhibit a ramified morphology and serve to support neuronal function and health via physical interactions and a vast repertoire of released signaling molecules and enzymes (Nimmerjahn et al., 2005; Kierdorf and Prinz, 2017; Hume et al., 2019). Recent research indicates that these cells have a long lifespan, sustain their population through self-renewal, and display molecular and transcriptional diversity based on location within the CNS (Reu et al., 2017; Masuda et al., 2019; Zhan et al., 2019). When faced with infections, aging, or injuries, microglia adapt in terms of transcription, morphology, and function; changes that are typically beneficial or vital for recovery (Hammond et al., 2019; Mathys et al., 2019). In acute inflammatory events, the pro-inflammatory response resolves and microglia continue their surveillance of the brain parenchyma, returning to brain homeostasis. However, in response to injury or infection, the equilibrium between microglial surveillance and activation can be disturbed, creating a chronic neuroinflammatory state that can lead to tissue damage. Beyond their immune roles, it’s now understood that microglia play other roles in the homeostatic and developing brain (Salter Michael and Beggs, 2014; Li and Barres, 2018; Prinz et al., 2019). These findings have occurred across a backdrop of increasingly elegant methodological advances including single cell analyses (Hammond et al., 2019; Li et al., 2019; Masuda et al., 2019), microglial ablation paradigms (Elmore et al., 2014; Bruttger et al., 2015; Han et al., 2017, 2019; Green et al., 2020), and *in vivo* imaging techniques (Nimmerjahn et al., 2005; Miyamoto et al., 2016; Liu et al., 2019; Stowell et al., 2019), that together have characterized the dynamic influence microglia have on virtually all major CNS cell-types over the lifespan of an organism.

**FIGURE 1:**
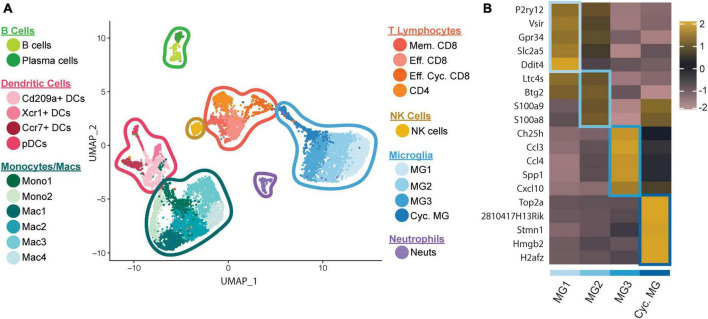
scRNAseq analysis of CD45+ cells within the CNS of JHMV-infected mice. C57BL/6 mice were infected i.c. with JHMV and brains collected at days 3 and 7 p.i. and spinal cords collected at day 21 p.i. **(A)** UMAP plot showing aggregate data of the immune landscape in brains and spinal cords of uninfected (control) and infected mice at 3, 7, and 21 days p.i. with JHMV revealing 22 distinct cell clusters (5–6 mice pooled per group). **(B)** Heatmap showing top 5 differentially expressed transcripts between heterogeneous subpopulations of microglia. Each heatmap is generated with subset data from microglia populations and isolated from all other clusters outside of what is shown in each individual map. Data are aggregated from uninfected (control) and infected at days 3, 7, and 21 p.i. Columns represent the different clusters, and rows represent expression of transcripts. Figures derived from Syage et al. (2020).

We recently employed scRNAseq on flow-sorted CD45+ cells enriched from the CNS of JHMV-infected mice at defined times to better understand these processes. We chose to examine brains during peak innate (day 3 p.i.) and adaptive (day 7 p.i.) responses, and spinal cords to evaluate immune cell responses during chronic demyelination (day 21 p.i.) (Syage et al., 2020). Downstream analysis of processed data on cells isolated from brains and spinal cords of control and infected mice at days 3, 7, and 21 p.i., revealed 22 unique clusters (Figure 1A). With regard to the four microglia subsets (MG1, MG2, MG3, and Cycling MG), each had unique transcriptional signatures enabling grouping into specific subsets, further enforcing the notion that microglia exhibit dynamic responses following JHMV infection of the CNS (Figure 1B).

**FIGURE 2:**
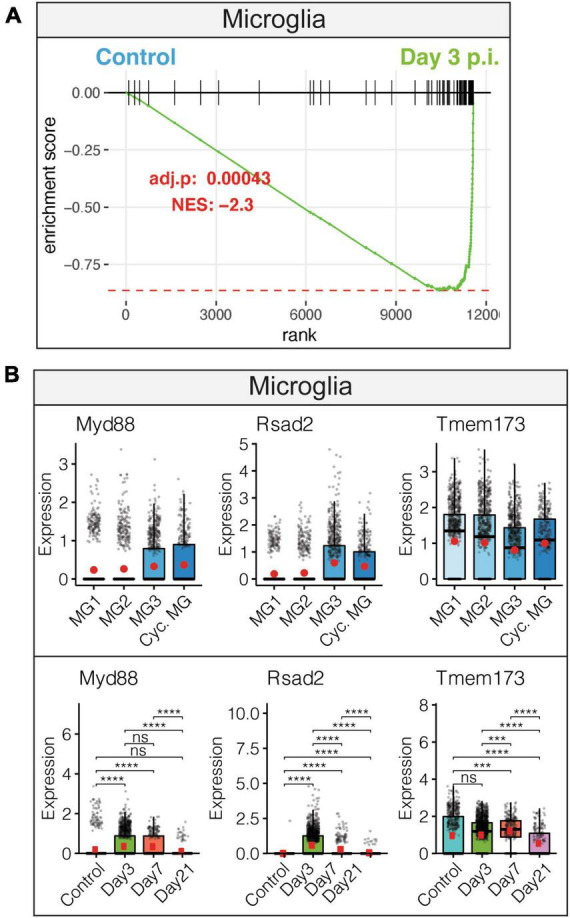
Microglial innate immune response following JHMV infection of the CNS. **(A)** Gene set enrichment analysis (GSEA) for IFN-α responses in microglia at day 3 p.i. from brains of JHMV-infected mice. Area under the curve represents enrichment of response genes. Normalized enrichment scores and *p*-values shown. **(B)** Data showing expression of *Myd88*, *Rsad2*, and *Tmem173* transcripts in microglia subpopulations as well as overall temporal expression at defined times p.i. with JHMV. Box plots show interquartile range, median value (bold horizontal bar), and average expression value per sample (red dots). ns, not significant, ***p < 0.001, ****p < 0.0001. Images derived from Syage et al. (2020).

Examination of innate anti-viral immune responses by microglia following JHMV infection revealed increased interferon alpha (IFN-α) responses. This observation was accompanied by increased expression of transcripts encoding anti-viral response factors including *Myd88*, *Rsad2* (Viperin), and *Tmem173* (STING) that were enriched in distinct subpopulations of microglia (Figures 2A, B). Expression of anti-viral effector responses at early stages of JHMV infection appear regulated within different subpopulations of microglia and this may reflect response to viral infection within discrete anatomic locations within the brain and/or exposure to type I interferons that can affect expression of anti-viral transcripts.

**FIGURE 3:**
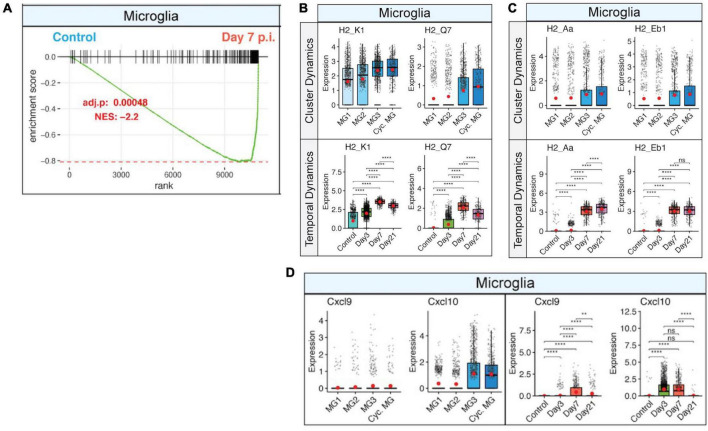
IFN-γ signaling activates microglia and corresponds with increased expression of MHC class I and II transcripts. **(A)** Gene set enrichment analysis (GSEA) for IFN-γ responses in microglia at day 7 p.i. from brains of JHMV-infected mice. Area under the curve represents enrichment of response genes. Normalized enrichment scores and *p*-values shown. Box plots showing expression of **(B)** MHC class I-associated genes, *H2-K1* and *H2-Q7*, and **(C)** expression of MHC class II-associated genes, *H2-Aa* and *H2-Eb1*, in subpopulations of microglia in control mice and at defined times p.i. **(D)** Expression of *Cxcl9* and *Cxcl10* transcripts in microglia (MG) subsets in control mice and at defined times p.i. Box plots show interquartile range, median value (bold horizontal bar), and average expression value per sample (red dots). ns, not significant, **p < 0.01, ****p < 0.0001. Images derived from Syage et al. (2020).

Viral replication peaks between 5 and 7 days p.i., and this is accompanied by a robust inflammatory response within the CNS. At the same time, there is a decline in viral replication that is associated with infiltration of virus-specific CD4+ and CD8+ T cells expressing IFN-γ and perforin (Bergmann et al., 2004). Microglia are responsive to IFN-γ signaling (Figure 3A), which enhances expression of MHC class I-associated transcripts (Figure 3B) and MHC class II-associated transcripts (Figure 3C). Temporal analysis of these transcripts in microglia show very low expression in uninfected mice that gradually increases by day 3 p.i. and is dramatically elevated by days 7 and 21 p.i (Figures 3B, C). JHMV infection invokes expression of T cell chemoattractant chemokines, CXCL9 and CXCL10, that attract activated T and B lymphocytes into the CNS via binding to the receptor CXCR3; this is central in promoting an effective host defense by controlling viral replication (Liu et al., 2001a,b, c; Phares et al., 2013). While astrocytes are important sources of both CXCL19 and CXCL10 in response to CNS infection by JHMV (Lane et al., 1998; Liu et al., 2001a; Phares et al., 2013), microglia subsets also express these transcripts within the brain at days 3 and 7 p.i. but to a lesser extent within the spinal cord at day 21 p.i (Figure 3D; Syage et al., 2020). We interpret these findings to indicate that microglia exert an important role in host defense following JHMV infection during acute disease by aiding in attracting virus-specific T cells into the CNS and subsequently presenting viral antigen. During chronic immune-mediated demyelinating disease, microglia may play a more subordinate role to astrocytes and monocyte/macrophages with regards to expressing T cell chemoattractants.

We have also determined that commensals aid in host defense following JHMV infection of the CNS through enhancing microglia function (Brown et al., 2019). Germfree mice or animals that receive antibiotics are unable to control JHMV replication within the brain during acute disease. The impaired ability of virus-specific T cells in germfree mice to control viral replication was not due to T cell-intrinsic defects but was the result of deficient MHC class II expression on microglia. Moreover, oral administration of toll-like receptor (TLR) to JHMV-infected mice limited the severity of clinical disease, and this correlated with increased MHC class II expression on microglia and efficient control of viral replication within the brains. Further work revealed that signaling through TLR4 is important in the homeostatic activation of microglia, as targeted genetic disruption of *Tlr4* within microglia leads to increased viral-induced clinical disease (Brown et al., 2019). These findings demonstrate that gut immune-stimulatory products can influence microglia function to prevent CNS damage following viral infection.

**FIGURE 4:**
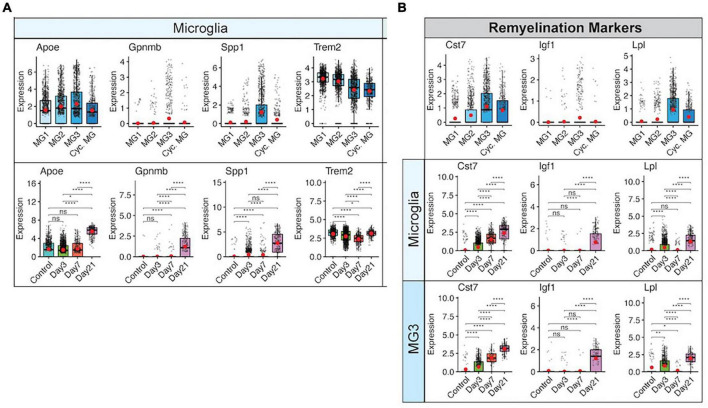
Microglia contribute to JHMV-induced demyelination and promote remyelination. **(A)** Expression of transcripts, *Apoe*, *Gpnmb*, *Spp1*, and *Trem2*, that are associated with demyelination are elevated in spinal cords at day 21 p.i. in specific subpopulations of microglia in control mice and at defined times p.i. **(B)** Expression of remyelination markers, *Cst7*, *Igf1*, and *Lpl*, are also increased in specific subpopulations of microglia in spinal cords at day 21 p.i. with JHMV. Box plots show interquartile range, median value (bold horizontal bar), and average expression value per sample (red dots). ns, not significant, *p < 0.05, **p < 0.01, ****p < 0.0001. Images derived from Syage et al. (2020).

By day 21 p.i., JHMV persists within the spinal cords of surviving mice, and this is associated with ongoing neuroinflammation and demyelination in which inflammatory T cells and monocytes/macrophages contribute to white matter damage (Wu and Perlman, 1999; Wu et al., 2000; Glass et al., 2004). Microglia express transcripts encoding genes associated with demyelination including Apolipoprotein E (*Apoe*), Transmembrane glycoprotein NMB (*Gpnmb*), Osteopontin (*Spp1*), and Triggering receptor expressed on myeloid cells 2 (*Trem2*) (Chabas et al., 2001; Ulrich and Holtzman, 2016; Hendrickx et al., 2017; Krasemann et al., 2017; Figure 4A). Under non-disease conditions, microglia function to maintain tissue homeostasis and have also been shown to be important in maintaining overall myelin health through regulation of myelin growth as well as preservation of myelin integrity (Yin et al., 2017; Amor et al., 2022; McNamara et al., 2023). Following JHMV-induced demyelination, microglia subsets express transcripts encoding markers associated with remyelination, including cystatin F (*Cst7*) (Ma et al., 2011; Shimizu et al., 2017), insulin-like growth factor 1 (*Igf1*) (Ye et al., 2002; Wlodarczyk et al., 2017), and lipoprotein lipase (*Lpl*) (Bruce et al., 2018; Figure 4B). The highest levels of expression for *Cst7*, *Igf1*, and *Lpl* occurred within the spinal cord at day 21 p.i. Additionally, microglia express transcripts associated with phagocytosis of myelin debris at both days 14 and 21 p.i. and electron micrographs show microglia containing phagocytosed myelin and axonal debris at day 21 p.i. (Sariol et al., 2020), a function that is essential for remyelination to occur. These findings support the notion that microglia may either directly or indirectly influence remyelination within the spinal cord by contributing to expression of genes encoding proteins that regulate oligodendrocyte precursor cell (OPC) survival and maturation (Lloyd and Miron, 2019; Lloyd et al., 2019).

## 3 Pharmacologic ablation of microglia impacts effective T cell-mediated control of JHMV replication within the CNS

The functional roles of microglia in contributing to host defense in response to CNS infection with neurotropic viruses have been greatly elucidated by findings demonstrating that mice lacking colony stimulating factor 1 receptor (*Csfr1-/-*) lack microglia, emphasizing the importance of this signaling pathway in microglia development (Ginhoux et al., 2010). Subsequent studies by Elmore et al. (2014) showed that blocking CSF1R signaling in adult mice through administration of CSF1R antagonists leads to > 95% reduction in microglia, demonstrating that CSF1R is necessary for microglia survival. More recently, treatment of mice with PLX5622, a brain penetrant and selective antagonist of the CSF1R that results in a dramatic reduction in microglia, has helped elucidate the functional roles of these cells in pre-clinical models of neurodegenerative disease (Elmore et al., 2014; Dagher et al., 2015; Acharya et al., 2016; Spangenberg et al., 2019). Wheeler et al. (2018) were the first to demonstrate that administration of PLX5622 prior to JHMV-infected mice resulted in increased mortality, associated with impaired control of viral replication within the brain during early stages of disease. Increased viral titers in PLX5622-treated mice were associated with a reduction in MHC class II expression on monocytes/macrophages similar to findings by Wheeler et al. (2018). Subsequent studies showed that PLX5622-mediated ablation of microglia results in increased susceptibility to West Nile virus (Seitz et al., 2018; Funk and Klein, 2019), Japanese encephalitis virus (JEV) (Seitz et al., 2018), and Theiler’s murine encephalomyelitis virus (TMEV) (Waltl et al., 2018; Sanchez et al., 2019), further supporting a protective role for microglia against acute viral-induced encephalitis.

Our laboratory depleted microglia via administration of PLX5622 to mice prior to JHMV-infection to further evaluate how microglia tailor the immune environment in response to CNS infection (Mangale et al., 2020). We employed scRNAseq to better understand how depletion of microglia within the CNS influenced transcriptional responses by myeloid cells and infiltrating lymphocytes. Similar to Wheeler et al. (2018), our findings indicated that depletion of microglia prior to JHMV infection resulted in increased mortality, associated with higher viral titers within brains and spinal cords. Expression of type I interferons (IFN) is a critical first line of defense that limits viral dissemination, and microglia are considered important sentinel cells capable of type I IFN expression (Ireland et al., 2008). We found that ablation of microglia led to increased expression of IFN-α in both macrophages and dendritic cells, suggesting that these cells were responding to the overall increased levels of virus within the CNS. Although we did not examine expression of type I IFNs in other cells types, it is also reasonable to argue that resident CNS cells, including neurons and astrocytes, were also able to respond to elevated levels of virus through production of type I IFNs (Hwang and Bergmann, 2018, 2019, 2020). There was not a dramatic change in immune cell infiltration into the CNS of JHMV-infected, PLX5622-treated mice compared to control mice, arguing that microglia may not be critical in terms of impacting neuroinflammation in response to infection. However, CD4+ T cells from PLX5622-treated mice did exhibit a reduced activation state, as determined by a reduction in expression of transcripts encoding Th1-associated transcription factor, *Tbet*, and activation markers, *Cd69* and *Cd44*. This overall muted CD4+ T cell response was associated with a reduction in expression of MHC class II transcripts and protein by macrophages within the brains of PLX5622-treated mice. In contrast, there were no differences in expression of MHC class I expression on macrophages in PLX5622-treated mice, and expression of both MHC class I and II transcripts in dendritic cells was not affected. Collectively, these findings argue that during acute disease, microglia tailor the CNS microenvironment in response to JHMV infection by influencing effective host defense mechanisms that enable efficient T cell-mediated control of viral replication. Presumably, this is done through secretion of cytokines/chemokines that allow for efficient expression of MHC class II, promoting effective anti-viral CD4+ T cell responses, which subsequently enhance anti-viral CD8+ T cell responses.

## 4 Microglia restrict the severity of immune-mediated demyelination and influence remyelination

**FIGURE 5:**
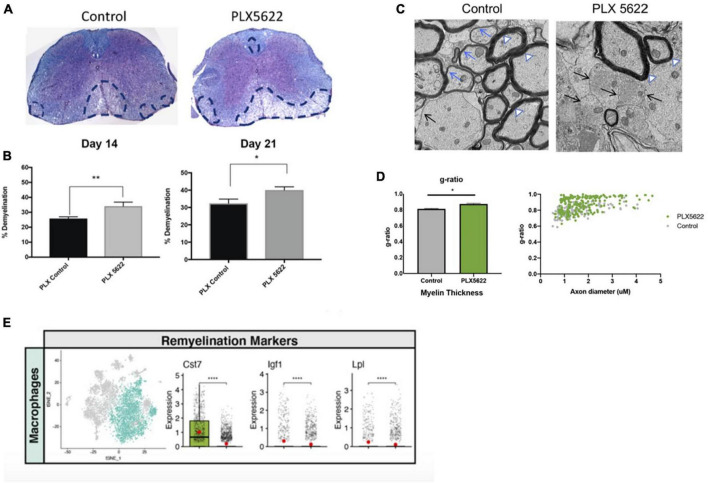
The severity of spinal cord demyelination is increased in PLX5622-treated mice. **(A)** Representative images of H&E/LFB-stained spinal cord sections showing an increase in severity of demyelination (dashed black lines) in JHMV-infected mice treated with PLX5622 compared to control treated mice at day 14 **(B)** Quantification of spinal cord demyelination reveals a significant increase after PLX5622 treatment compared to control-treated animals at days 14 and 21 p.i. **(C)** Representative EM images (1200×) from spinal cords from control and PLX5622-treated mice showing normal myelinated axons (white arrowheads), demyelinated axons (black arrows), and remyelinated axons (blue arrows) at day 21 p.i. **(D)** Calculation of *g*-ratio of control and PLX5622; scatter plot depicting individual *g*-ratios from lateral white matter columns of control (gray) and PLX5622 (green)-treated mice as a function of axon diameter. **(E)** t-SNE plots showing decreased expression of transcripts encoding remyelination-associated markers, *Cst7*, *Igf1*, and *Lpl* in spinal cords of PLX5622-treated mice compared to controls at day 14 p.i. *p < 0.05, **p < 0.01, ****p < 0.0001. Images derived from Mangale et al. (2020).

As part of our studies evaluating how PLX5622 treatment impacted host defense in response to CNS infection by JHMV, we also determined how microglia ablation affected the severity of demyelination. PLX5622 treatment led to increased spinal cord demyelination compared to control mice (Figures 5A, B). scRNAseq analysis indicated that the increase in white matter damage in PLX5622 was associated with increased expression of transcripts encoding molecules associated with demyelination including *Apoe* (Krasemann et al., 2017), *Spp1* (Chabas et al., 2001), and *Trem2* (Ulrich and Holtzman, 2016; Krasemann et al., 2017) in macrophage clusters. In addition, electron microscopy (EM) revealed impaired remyelination in PLX5622-treated mice as indicated by an increase in the *g*-ratio (the ratio of the inner axonal diameter to the total outer fiber diameter) (Figures 5C, D). There was a reduction in expression of transcripts associated with remyelination including *Cst7*, *Igf1*, and *Lpl* in spinal cord macrophages of PLX5622-treated mice (Figure 5E; Ye et al., 2002; Ma et al., 2011; Hlavica et al., 2017; Shimizu et al., 2017; Wlodarczyk et al., 2017; Bruce et al., 2018; Durose et al., 2019). This suggests that microglia impacted the expression of molecules associated with both demyelination and remyelination in macrophages.

Determining how targeted ablation of microglia influences the microenvironment once JHMV-induced demyelination is already established is another important and clinically relevant question for potentially treating a number of neurodegenerative diseases. Elegant studies by Sariol et al. (2020) demonstrated that treatment with PLX5622 at various times following JHMV resulted in varied outcomes in host defense, demyelination, and remyelination. For example, depletion of microglia beginning at day 7 p.i. with JHMV resulted in worsened clinical disease, associated with increased demyelination and reduced remyelination, yet CNS viral titers were not affected when compared to control mice. Consistently, ablation of microglia during acute disease (day 7 p.i.) led to increased accumulation of extracellular vesiculated myelin and cellular debris in the spinal cords of PLX5622-treated mice, combined with demonstrated differential expression of genes involved in myelin debris clearance. In contrast, PLX5622-treatment beginning at day 15 p.i. did not affect the severity of clinical disease nor increase demyelination. Subsequently, we published findings indicating that PLX5622-mediated depletion of microglia beginning at day 14 p.i led to an increase in the severity of demyelination and impaired remyelination compared to control mice. As well, gene expression analysis revealed that ablating microglia resulted in altered expression of genes associated with immune cell activation and phagocytosis of myelin debris (Cheng et al., 2023). Collectively, these findings argue that PLX5622 treatment following JHMV infection does affect the severity of both demyelination and remyelination. This indicates that microglia continue to exert an important role in influencing neuropathology during acute/sub-acute stages of disease, and phagocytosis of myelin debris specifically by microglia is important in regulating tissue damage and repair.

## 5 Human coronaviruses and neurologic disease

Coronaviruses that have been identified that infect humans (HCoV’s) include HCoV-229E, HCoV-OC43, HCoV-NL63, HCoV-HKU1, severe acute respiratory syndrome (SARS)-CoV, Middle East respiratory syndrome (MERS)-CoV, and SARS-CoV-2. Notably, infection with all of these viruses has been associated with effects on neurologic function (Morgello, 2020). The four commonly circulating HCoV’s, 229E, OC43, NL63, and HKU1, are often referred to as the common cold coronaviruses and account for approximately 15–20% of seasonal colds and are associated with mild-to-moderate infections of the upper respiratory tract (Holmes and Lai, 1996; Perlman et al., 1999; Wang et al., 2020). Comparatively, SARS-CoV-1, MERS-CoV, and SARS-CoV-2 have been associated with severe respiratory disease and have prompted public health emergencies. In addition to severe respiratory disease following infection with these viruses, CNS invasion and neuropathology have been reported (Iadecola et al., 2020). In comparison with SARS-CoV-1 and MERS-CoV, neurologic symptoms which range in severity are more common following SARS-CoV-2 infection. Neuropathological findings associated with SARS-CoV-2 infection include lymphocyte inflammation, acute hypoxic-ischemic changes, astrogliosis, and spontaneous hemorrhage (Pleasure et al., 2020; Lou et al., 2021; Maury et al., 2021; Schwabenland et al., 2021; Stein et al., 2023). In addition, microglial activation is also commonly detected in COVID-19 patients (Pleasure et al., 2020; Lou et al., 2021; Maury et al., 2021; Schwabenland et al., 2021; Stein et al., 2023). SARS-CoV-2 viral RNA and antigen have been detected within the CNS and cerebral spinal fluid of COVID-19 patients upon post-mortem analysis. This finding, along with the observation that the virus is able to infect and replicate within resident cells of the CNS, supports the view that the virus is neurotropic (Matschke et al., 2020; Bellon et al., 2021; Olivarria et al., 2022; Proust et al., 2023). Neurologic symptoms associated with COVID-19 are thought to occur via a variety of different mechanisms, including endothelial damage of the blood-brain-barrier (BBB), which is associated with increased capillary damage (Helms et al., 2020; Magro et al., 2020). While cytokine storm is considered to be a major cause of acute respiratory distress syndrome (ARDS) and multiple organ failure (Chousterman et al., 2017), it also is considered to be a contributing factor of neurological complications of COVID-19 (Almutairi et al., 2021; Wang and Perlman, 2022). It has been observed that patients with more severe COVID-19 have a more drastic inflammatory immune response (Chen et al., 2020; Huang et al., 2020), leading to the release of proinflammatory cytokines (Huang et al., 2020; Ragab et al., 2020; Ye et al., 2020). Some of these inflammatory markers including IL-6 and IL-1β are also elevated in animal models (Klein et al., 2021) and are associated with impaired neurogenesis and hippocampal dependent memory (Garber et al., 2018; Kong et al., 2019). IL-6 is associated with increased viral loads and disease severity (Chen et al., 2020, 2021) and has been demonstrated to be involved in neurodegenerative diseases mediated by neuroinflammation (Strafella et al., 2020). Increased cytokine levels have also been seen in patients experiencing pneumonia and hypoxia (Arnaldez et al., 2020), and hypoxic changes *in vitro* and in patients have been associated with neuronal death and loss (Song et al., 2021; Solomon and Liang, 2022). In addition, evidence of neuronal degeneration and changes in glial cell morphology are also present in some hippocampal tissues derived from COVID-19 patients (Bayat et al., 2022). As indicated above, microglia have been shown to have distinct roles in host defense and disease in response to infection of the murine CNS with JHMV; similarly, microglia are suspected to have some role in neurologic manifestations associated with COVID-19 as well as long COVID (also called Post-Acute Sequelae of COVID, PASC) (Schwabenland et al., 2021; Albornoz et al., 2022; Jeong et al., 2022; Monje and Iwasaki, 2022; Strong, 2023; Thaweethai et al., 2023; Wei et al., 2023). Moreover, animal models of SARS-CoV-2 infection have demonstrated microglial activation and a role in the expression of cytokines/chemokines associated with neuroinflammation (Munoz-Fontela et al., 2020; Amruta et al., 2022; Olivarria et al., 2022). Ongoing studies involving COVID-19 patients as well as pre-clinical animal studies will ultimately reveal how microglia and other CNS resident cells affect neurologic disease in SARS-CoV-2-infected individuals.

## 6 Perspectives

**FIGURE 6:**
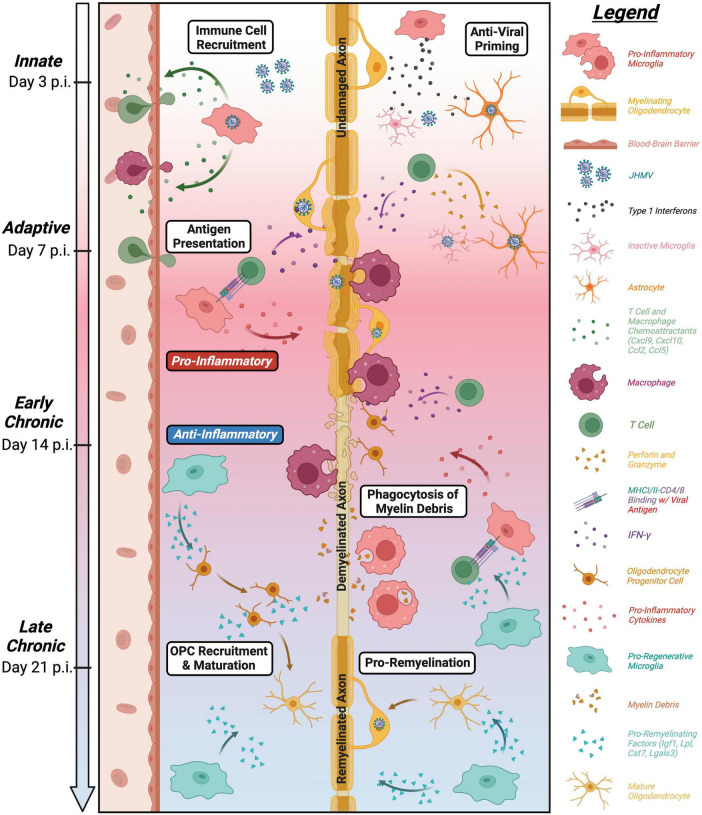
Roles of microglia in defense, disease, and repair in response to JHMV infection of the CNS. Overview of influence of microglia at defined stages following CNS infection of the CNS. Microglia aid in defense on at innate and adaptive stages of infection through release of type I interferons along with other antiviral pathways as well as antigen presentation to virus-specific T cells (Wheeler et al., 2018; Mangale et al., 2020). While these anti-viral responses help control viral replication, JHMV persists in white matter tracts resulting in chronic inflammation and demyelination. Microglia can secrete pro-inflammatory cytokines/chemokines that attract inflammatory T cells and myeloid cells that amplify white matter damage yet microglia may also restrict the severity of demyelination potentially by regulating expression of disease-associated genes in other myeloid cells (Mangale et al., 2020; Sariol et al., 2020; Syage et al., 2020). In addition, recent evidence supports a role for microglia in restricting the severity of demyelination as well as promoting remyelination through phagocytosis of myelin debris and secretion of factors that promote OPC recruitment/survival and maturation into mature myelin-producing oligodendrocytes (Lloyd et al., 2019; Sariol et al., 2020; Syage et al., 2020; Cheng et al., 2023).

It is now well-recognized that, in addition to serving as the immune sentinel cell of the brain, microglia exert key roles in CNS development, tissue homeostasis, and response to both injury and infection. These diverse roles of microglia have been clearly revealed within the context of viral infection of the CNS. The JHMV model of viral-induced encephalomyelitis and immune-mediated demyelination has provided important insight with regards to how microglia influence host defense during acute disease, as well as how these cells participate in restricting demyelination and enhancing remyelination. An overview of roles of microglia in distinct stages of defense and disease in response to JHMV infection of the CNS is provided in Figure 6. Ongoing studies, using increasingly sophisticated approaches, are required to gain additional insight into the molecular mechanisms by which microglia impact the microenvironment and influence resident CNS cells and inflammatory cells to effectively respond to viral infection and aid in tissue recovery, with the ultimate goal of identifying unique therapeutic targets that may aid in dampening disease progression in human demyelinating diseases as well as individuals infected with human coronaviruses.

## Author contributions

AS: Conceptualization, Writing – original draft, Writing – review & editing. CP: Conceptualization, Writing – original draft, Writing – review & editing. YC: Writing – review & editing. VM: Writing – review & editing. KG: Writing – review & editing. TL: Funding acquisition, Project administration, Writing – original draft, Writing – review & editing.
